# Design Guidelines for a Game-Based Physical Rehabilitation System: Focus Group Study

**DOI:** 10.2196/67336

**Published:** 2025-09-26

**Authors:** Ahmad M S Elaklouk, Ratna Zuarni Ramli, Samah M M Alakklouk, Norsafinar Rahim

**Affiliations:** 1 School of Computing and Informatics, Universiti Teknologi Brunei, Jalan Tungku Link Gadong, Bandar Seri Begawan, BE1410, Brunei Darussalam, 673 2461020, 673 2461035; 2 Universiti Teknologi MARA Cawangan Negeri Sembilan Kampus Seremban, Seremban, Malaysia; 3 Centre for Instructional Technology and Multimedia, Universiti Sains Malaysia, Penang, Malaysia

**Keywords:** serious games, physical rehabilitation, game design, brain damage, kinect, game, gamification, therapy, therapist, practitioner, game-based, physical, rehabilitation, virtual reality, VR, movement, motor

## Abstract

**Background:**

Incorporating serious games and advancements in information and communication technologies into physical rehabilitation can substantially enhance the process, provide unique benefits, and improve its effectiveness and efficiency. While recent literature highlights various game-based interventions for physical rehabilitation, there is a lack of comprehensive guidance on how to design and develop systems that effectively address the actual needs of therapists, practitioners, and individuals with physical disabilities.

**Objective:**

The objective of this study was to explore the intentions, needs, and desires of therapists and other practitioners, as well as to examine the factors and determinants influencing the effectiveness and efficacy of game-based physical rehabilitation, since therapists and other health care practitioners play crucial roles in both patient recovery and the establishment of an effective game-based therapy.

**Methods:**

A design science approach was adopted to achieve this research objective. A focus group of 27 participants was conducted to gather feedback, identify user needs, and understand the requirements for game-based physical rehabilitation. The participants first tested commercially available games and then evaluated mock-ups of the proposed game prototypes.

**Results:**

This study provides essential design insights and guidelines for designers and researchers, focusing on the practical needs and requirements of game-based physical rehabilitation systems.

**Conclusions:**

As proof of concept, these guidelines will be used in the next phase of our research, which involves designing and developing a game-based physical rehabilitation system.

## Introduction

The application of technology in rehabilitation is experiencing significant and rapid expansion [1]. The integration of serious games and technological advancements enhances the rehabilitation process, creating new opportunities to address stakeholders’ needs and expectations by (1) enhancing the quality and efficacy of rehabilitation, (2) offering alternatives that sustain patient motivation by mitigating the repetitive nature of traditional rehabilitation, (3) providing tailored therapy levels, and (4) overcoming limitations in resources and facilities associated with conventional rehabilitation methods [2-13]. For example, individuals who have experienced a stroke are motivated to use technologies and games to facilitate their functional recovery [14]. Furthermore, the study by Cruz et al [15] revealed that commercial gaming consoles and off-the-shelf games are more affordable and offer numerous options and variations, which could facilitate the implementation of virtual reality games in clinical practice. However, the usability of virtual reality games is a crucial factor to consider in the design of these interventions. The study by Daoud et al [16] introduced a game-based rehabilitation system that includes 3 exercises and a computerized assessment method. This system evaluates the accuracy of right arm movements during gameplay by analyzing tracking data collected from a Microsoft Kinect sensor. The study by Jayasree-Krishnan et al [17] discussed several barriers to the seamless integration of technological solutions into clinical care, including issues with accessibility to quality rehabilitation, adaptability to individual patient differences, compliance, and engagement with the rehabilitation process.

In recent years, the implementation of diverse technologies has strengthened telerehabilitation, enhancing rehabilitation processes and ensuring patients receive the necessary services [11,18,19]. Integrating physiotherapy with technologies such as virtual reality and video games enhances the rehabilitation of poststroke patients compared to physiotherapy alone. This approach offers an immersive and interactive platform that boosts patient engagement and motivation during rehabilitation, while also providing a safe environment for practicing and improving physical abilities [2,20,21]. Evidence from a range of studies highlights its potential to improve outcomes for individuals with various physical impairments [2]. Takei et al [22] assessed the safety, feasibility, and acceptability of using the Nintendo Switch video game Ring Fit Adventure for upper and lower limb strength training in older adults with musculoskeletal conditions, alongside conventional physiotherapy over 6 sessions. The authors concluded that this approach is a safe and feasible adjunct to a rehabilitation program, as long as it is supervised, particularly in the initial stages. In the study by Wittmann et al [23], the game “Meteors” was introduced, which involves a virtual robotic arm that replicates the player’s arm movements to catch meteors falling onto a planet. In the study by Wittmann et al [24], the game “Slingshot” was used to enhance arm coordination and improve the precision of aiming and extending arm movements. This game focuses on exercising the flexion and extension of the elbow. In this game, scoring is based on performance, and the difficulty level can be dynamically adjusted to maintain the patient’s motivation and commitment. In the study by Goršič et al [25], 4 video games were developed for arm rehabilitation. One game involved competitive play, where the patient competed against another individual, such as a friend, relative, or therapist. In addition, there were 2 cooperative games, where the patient and another player worked together to play against the computer, and 1 single-player game where the patient played alone against the computer. The study revealed that competitive games significantly enhanced functional recovery and improved patients’ quality of life more than conventional rehabilitation exercises.

In the study by Ma et al [26], a full-body rehabilitation video game was developed using Kinect V2 as an input device. The game accommodates both sitting and standing positions, supporting various movements such as gross motor actions (steps, jumps, and squats) and fine motor gestures (handshakes, palm rotations, and hand opening and closing). This system tracks the player in 3D space and registers real-time data, demonstrating positive outcomes in motor function and the performance of basic daily activities among chronic poststroke patients. The study by [19] argued that successful games in the industry, known for their immersive qualities, incorporate 8 key features: game control technology, display technology, rewards, social elements, avatars, game difficulty, music or sound, and graphical realism. They proposed that future research could explore different game genres, aspects like game narrative and competition, improvements in user interface, and the integration of immersive technologies such as virtual and augmented reality.

The exploration of therapeutic alternatives, combined with new technologies, enables professionals to use a variety of tools for patient rehabilitation and treatment. However, it is crucial to comprehend the functionalities, potential applications, and limitations of these tools [27]. Future research should aim for higher methodological quality, larger sample sizes, and well-defined rehabilitation programs to mitigate inconsistencies in evidence within this field [3,28].

Video games designed for therapeutic use have distinct requirements compared to regular video games. There are differences in user characteristics, where the patient serves as the primary player and the therapist as a secondary user, in contrast to players of regular video games. These users also have different objectives, and the context varies [27]. Considering the patient as the sole end user for any rehabilitation intervention is an aberration and diverges from standard practice, as therapists are primarily responsible for motivating, guiding, and assessing the patient. The study by Krishnan et al [14] revealed that, although individuals who have experienced a stroke were amenable to integrating exergames into their rehabilitation regimen, they noted that these games could not replace the essential role of therapist supervision. Integrating new technologies into clinical practice is complex, as it requires addressing the needs of both clients and clinicians. By considering these factors, technology developers can improve the chances that clinicians will embrace and adopt innovative technologies [29].

The study by Hamilton et al [30] highlighted 2 key factors for optimizing patient engagement: adequate support and a perceived benefit from the technology. It concluded that patients are more likely to engage with technology during rehabilitation when it is personalized by a therapist to fit their specific needs. Therapists and other health care practitioners play crucial roles in both patient recovery and the establishment of an effective rehabilitation system [31,32].

Since rehabilitation aims to restore patient independence and enhance the quality of life, achieving optimal outcomes requires a cohesive multidisciplinary team comprising doctors, psychologists, occupational therapists, nurses, social workers, and physiotherapists collaborating effectively [27]. Despite numerous studies on technology acceptance, there is still a lack of understanding regarding the factors that influence health care professionals’ adoption of technological innovations [27,33]. The study by Jung et al [34] argued that although there has been speculation about the roles of therapists in game-based therapy and suggestions regarding varying levels of patient engagement, no comprehensive studies have been conducted to investigate these matters in depth. Understanding how games influence and support the different roles of therapists remains an area for future study [34]. The study by Wu et al [35] argued that, although some serious games have been developed for physical therapy, little work has been conducted through a participatory design approach. The authors highlight the importance of involving diverse stakeholders in the design process to create more effective and user-centric serious games for rehabilitation. Therefore, effective implementation and use require collaboration from all parties involved in the process, including physicians, nurses, social workers, and other allied health professionals [27]. Thus, the objective of this study is to explore the intentions, needs, and desires of therapists and other practitioners, as well as to examine the factors and determinants influencing the effectiveness and efficacy of game-based physical rehabilitation.

## Methods

### Ethical Considerations

This study was conducted in accordance with ethical standards for research involving human participants. Ethics approval was obtained from the Ethics and Research Committee, Scientific Research Department, University College of Science and Technology, Gaza, Palestine.

All participants were provided with comprehensive information regarding the study’s purpose, procedures, and ethical considerations, enabling them to make an informed decision about their involvement. Informed consent was obtained from all participants, who were explicitly informed of their right to decline or withdraw from the study at any point without any repercussions. Participants’ personal information and responses were treated with strict confidentiality. Data were anonymized and stored securely, ensuring that individual identities could not be disclosed in any reports or publications.

### Study Design Overview

This study used a design science research methodology, encompassing 2 iterations. The first iteration investigated the therapeutic game requirements and proposed guidelines to guide developers and practitioners in the design of therapeutic games; this iteration will be documented in this paper. The second iteration will use the findings from the first iteration to construct the main artifact, which is a rehabilitation gaming system for physical rehabilitation. The earlier work outlines a more detailed description of the design science research methodology [36,37]. Therefore, in the first iteration, the limited expertise of therapists in using game-based physical rehabilitation for acquired brain injuries significantly prolonged the process of determining the appropriate research strategy. Hence, before any actual game development, conducting a focus group with therapists and related practitioners is a great starting point as it helps generate interesting discussions between physical therapists, occupational therapists, service providers, special educators, psychologists, game designers, software engineers, other practitioners, and the researchers. This process is crucial to understanding how rehabilitation exercises can be translated into a suitable game by identifying the required movements, constraints, difficulty levels, and outputs to design game interventions that align with these exercises. To achieve this objective, the first purpose of the focus group is to evaluate the low-fidelity game prototypes (paper mock-ups) developed and assess how well these prototypes meet the requirements of the rehabilitation exercises. These low-fidelity game prototypes were developed through collaborative team brainstorming sessions and an in-depth review of the literature [38-57]. The second purpose of the focus group is to evaluate the commercial games. The study by Pedraza-Hueso et al [58] determined the feasibility of using the Xbox Kinect gaming console (Kinect Adventure game series) in the rehabilitation of a single patient. This study highlights a usability gap, noting that further research is needed to assess the usability of the Xbox Kinect gaming system in clinical settings and across different patient populations. Whole-body motion capture VR allows a unique opportunity for individuals to experience a heightened sense of realism during task-specific therapeutic activities. However, clinicians need to be able to match a game’s components to an individual’s functional deficits [59]. Even though none of these games were designed for clinical purposes. The study by Rizzo and Kim [60] stated that “Designers of rehabilitation tasks can benefit from examining the formulas that commercial game developers use.” For example, the study by Burke et al [61], discussed that scoring mechanisms as well as mechanisms that maintain player engagement are commonly used in commercial games and can be used similarly in a rehabilitation setting. Therefore, testing commercial games such as the Kinect Adventure game series and Motionsports Adrenaline with therapists can also provide valuable insights into design, usability, and the various mechanisms used to motivate and maintain patient engagement. It can also help identify the missing components that make these games unsuitable for physical rehabilitation. Based on the focus group results, we can then move forward to create high-fidelity prototypes.

### Focus Group

**Figure 1.**
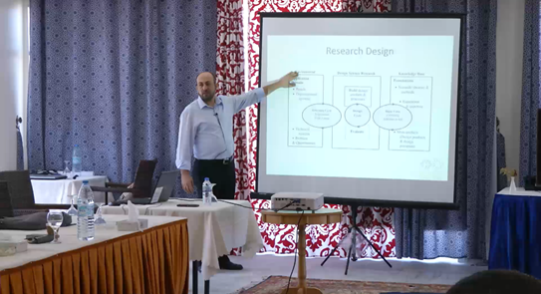
Focus group session briefing by the researcher.

**Figure 2.**
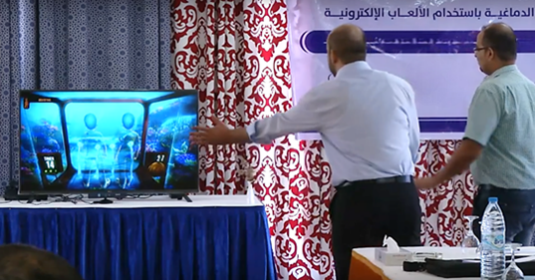
Focus group session. The participants are playing the Kinect Adventures game.

**Figure 3.**
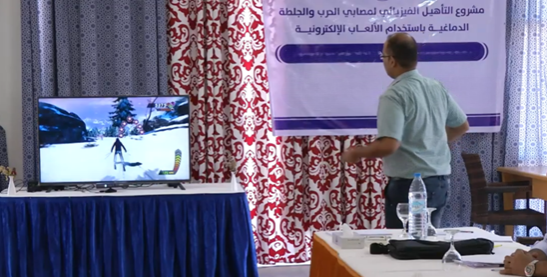
Focus group session. The participants are playing the Kinect Motionsports Adrenaline game.

The purpose of the focus group was to gather feedback from participants, explore their needs and expectations for a serious game, and gain insights into the requirements for game-based physical rehabilitation. The focus group (Figures 1-3) was held at a designated presentation venue in a hotel in Palestine and was divided into 2 sessions. The first session, lasting approximately 3.5 hours, involved the evaluation of selected commercial games. It began with an introductory briefing by the principal investigator, who outlined the study’s objectives. Research assistants initially facilitated the gameplay, helping participants become familiar with the interface. As the session progressed, some participants became comfortable navigating the game menus independently. Following the gameplay, an open discussion was conducted to gather participants’ feedback on their experiences and expectations. A scheduled break followed the conclusion of this session.

The second session, held after the break and lasting approximately 4 hours, focused on evaluating the game prototype mock-ups developed by the research team. The principal investigator presented each mock-up sequentially using a projector. Participants were then provided with structured questionnaires, which allowed them to share detailed feedback and offer suggestions for improvement. The completed questionnaires were collected by the research assistants for further analysis. Both sessions were audio- and video-recorded to support detailed qualitative data analysis.

### Participants

The focus group comprised 27 participants, including 4 occupational therapists, 10 physiotherapists, 2 game designers, 3 software engineers, 3 special educators, 3 psychologists, and 2 researchers. Table 1 shows the list of focus group participants, encompassing all participant categories.

**Table 1.** List of participants.

Role	Participant ID
Occupational therapists	P1-P4
Physiotherapists	P5-P14
Game designers	P15-P16
Software engineers	P17-P19
Special educators	P20-P22
Psychologists	P23-P25
Researchers	P26-P27

### Intervention Using Commercial Games

Motion sensing and tracking technologies, such as the high-performance 3D depth-sensing camera Astra Pro Plus, can be used in diverse applications, including robotics, serious games, virtual reality, and augmented reality. Motion-sensing devices, such as the Kinect, fall under the category of gesture-tracking devices, using a user interface known as a natural user interface. Consequently, participants moved around the play area, raised and lowered their arms, and adjusted their body positions to complete the tasks. A total of 2 commercially available games were selected: Kinect Adventures and Motionsports Adrenaline. Kinect Adventures was selected for its entertaining nature and its variety of mini-games, including Space Pop, 20,000 Leaks, Reflex Ridge, River Rush, and Rally Ball. These mini-games offer diverse scenarios and require different body movements. For instance, in 20,000 Leaks, players use their hands and feet to plug holes that appear when fish hit the tank glass. In Rally Ball, players hit a virtual ball toward targets, while in Reflex Ridge, they avoid obstacles while collecting coins. The second game chosen is Motionsports Adrenaline, which offers a range of sports experiences. It includes six distinct mini-games—Wingsuit, Kitesurfing, Kayaking, Mountain Biking, Rock Climbing, and Extreme Skiing—designed to simulate high-stakes stunts that most people would not attempt in real life. Unlike Microsoft’s Kinect Sports series, it stands out with a focus on realistic visuals, distinguishing it from many other Kinect games.

### Intervention Using Low-Fidelity Paper Mock-Ups

#### Rabbit Hunting

This game prototype displays several holes, with a rabbit hiding inside one of them. Once the game begins, the rabbit pops out of one of the 5 holes and stays in a hole for a short period of time (depending on the patient’s ability), as shown in Figure S1 in Multimedia Appendix 1. The player must hit the appearing rabbit with their hand to score a point. Using motion sensing devices, such as Kinect, the arm movement of the patient is mapped on the corresponding limb of the avatar (a model of a human represented on the screen). Feedback, such as a buzzing sound, an updated score counter, and a change in the rabbit’s color, is displayed to the patient on the screen. The game prototype aims to score as many points as possible within a specified time limit, which can be adjusted by the therapist according to the patient’s ability. The game becomes more challenging by increasing its speed or introducing a cat that the player must avoid. The therapeutic goals are to improve shoulder, elbow, and wrist movement; enhance multifunctional ability; develop eye-hand coordination; and strengthen muscle power.

#### Picking the Balls

The game prototype comprises three levels, each designed with increasing difficulty. The patient’s arm movements are tracked using motion-sensing devices, such as Kinect, and mirrored by a human avatar displayed on the screen. In the first level (see Figure S2 in Multimedia Appendix 1), the player simply places balls into a basket, with no requirement regarding color or order. Upon successful completion, the player advances to the second level (see Figure S3 in Multimedia Appendix 1), where colored balls are introduced. In this stage, the system prompts the player to pick a ball matching the color shown on the screen. In the final level (see Figure S4 in Multimedia Appendix 1), balls are marked with random numbers, and the player must place them into the basket in either ascending or descending numerical order. In the advanced levels, the patient will be asked which hand to use to pick up the balls. The therapeutic goal is to improve cognitive skills, endurance, eye-hand coordination, awareness, and the range of motion (ROM) of the arm.

#### Organizing Eggs

This prototype shows an egg appearing in the middle of the screen. Using motion sensing devices, such as Kinect, the avatar (the virtual character portraying the player on the screen) mimics the movements that the player makes. The player has to pick and put each egg in the specified place in the basket as shown in Figure S5 in Multimedia Appendix 1. The player gets a point for every egg put in the right place. The therapeutic goal is to improve motor functioning, balance, strengthen the muscle power in the whole upper limb, and movement of reach, grasp, and release.

#### Shuffling Shelves

This game prototype intends to work on the patient’s cognitive and physical capabilities, as the patient has to grab or pick the book from the upper shelf and put it on the lower one (see Figure S6 in Multimedia Appendix 1), depending on both the book and the shelf’s colors. The arm movement of the patient is mapped on the corresponding limb of the avatar (a model of a human represented on the screen) using the motion-sensing device recognition feature. The game begins with a series of colored books appearing on the upper shelf, above the divided and colored lower shelves. After the patient grabs the book and puts it on the suitable shelf, he gets a point. The therapeutic goals include improving endurance and balance, enhancing trunk control, developing right-left discrimination, refining the patient’s coordination skills, and promoting arm movements such as extension and shoulder abduction.

#### Hold the Boat

This prototype focuses on the arm’s strength and stability. Using the motion sensing device recognition feature, the avatar (the virtual character portraying the player on the screen) mimics the movements that the player makes. In this game prototype, the patient has to hold the boat and keep moving it to the goal (the end of the channel). Visual cues appear on the screen to guide the player, as shown in Figure S7 in Multimedia Appendix 1. The patient must not only reach the game objects (the boat) but also accompany their movements toward the final destination. This will promote the patient’s stability and persistence. The therapeutic goals are to improve grasping, hand awareness, stability, and shoulder ROM.

#### Reaping the Oranges

This game prototype includes 2 modes: single-player and multiplayer. In the single mode, the patient plays alone, collecting as many oranges as possible from the tree during a certain period of time. Using the motion sensing device recognition feature, the avatar (the virtual character portraying the player on the screen) mimics the movements that the player makes. The player collects the oranges by simply moving their hands to touch the orange on the tree, then placing it in the baskets displayed on the screen, as shown in Figure S8 in Multimedia Appendix 1. The game ends when the time limit is reached. The patient’s score is displayed, which is the number of oranges collected during the game. In the multiplayer mode, the goal of the game does not change, but there are two options: competition or collaboration. In the competition, both participants (either 2 patients or a patient and a therapist) must collect as many oranges as possible. The winner will be the one who reaps the most oranges. In the collaboration, both participants must collect as many oranges as possible and place them in shared baskets. The game ends when the time limit is reached or when they reach the target number of oranges, which can be set and controlled by the therapist. In the next game levels, the patients can be asked to use either their right or left arm to place the oranges in the right or left basket, depending on their condition. The therapeutic goal is to strengthen the upper limb, improve eye-hand coordination, range of motion for the arm, “Right-Left” discrimination, weight shifting, abduction, and adduction of the upper limbs of patients, and social interaction. In the next two game prototypes, the objective remained the same as described above, with the difference that the game objects the player must touch would be moving rather than static. This adds complexity, promoting stability and persistence.

#### Ice Balls

In this game prototype, the patient has to move his hands (stretching his arms at different lengths) to catch ice ball drops from the top of the screen as shown in Figure S9 in Multimedia Appendix 1. The game begins with a small number of ice balls falling slowly from the top of the screen. The arm movement of the patient is mapped on the corresponding limb of the avatar (a model of a human represented on the screen) using the motion-sensing device recognition feature. The more ice balls a player catches, the higher the score is. In the next levels, the number and speed of dropping ice balls increased, and in more difficult places to reach. The therapeutic goal is to improve upper limb movement and coordination, motor planning, timing of reaching, and the supination and pronation movement of the forearm.

#### Blowing the Bubbles

In this game prototype, the player must move their hand (or both hands). Using the motion sensing device recognition feature, the avatar (the virtual character portraying the player on the screen) mimics the movements that the player makes. The game starts with a girl on the side of the screen generating bubbles, and the player starts collecting points by blowing those bubbles with his or her hand as shown in Figure S10 in Multimedia Appendix 1. The game gets harder by creating more bubbles and making them faster and harder to blow. The therapeutic goal is to improve supination and pronation movements of the forearm, as well as shoulder and elbow muscle power and elbow ROM.

#### Matching Shapes

This game prototype focuses on cognitive and movement skills. The game starts as several shapes appear randomly on the screen (see Figure S11 in Multimedia Appendix 1). The arm movement of the patient is mapped on the corresponding limb of the avatar (a model of a human represented on the screen) using the motion sensing device recognition feature. The patient must use both arms to select matching shapes or identical symbols. The earlier levels have fewer and simpler shapes, while the advanced levels present more complex shapes. The player gets a point for correctly matching shapes. The therapeutic goal is to improve bilateral hand functioning, movement for the whole upper limb, coordination, and depth perception.

#### Colored Boxes

This game prototype focuses on cognitive and movement rehabilitation. The arm movement of the patient is mapped on the corresponding limb of the avatar (a model of a human represented on the screen) using the motion-sensing device recognition feature. The patient uses both upper limbs to transport boxes from one side to the other. On the left of the screen, the colored boxes (red, yellow, and blue) gradually depart from one of the 3 levels (the moving belts). The player stands in the middle of the screen using his upper extremities to make a bridge connecting both sides so that the appropriate colored box moves towards the same truck color as shown in Figure S12 in Multimedia Appendix 1. As the game levels advance, the speed of the colored boxes increases. The therapeutic goal is to improve bilateral hand functioning, endurance, coordination of movement in the upper limb, and hand control.

#### Fallen Apples

In this prototype, the patient raises their hands and moves them left and right, causing the apples to drop, as shown in Figure S13 in Multimedia Appendix 1. Using the motion sensing device recognition feature, the avatar (the virtual character portraying the player on the screen) mimics the movements that the player makes. As the game begins, the trees appear with apples on them, and the player starts moving their hands left and right. A correct movement causes the apples to drop, earning the patient a point. As the levels increase, the difficulty also rises, requiring the player to move their arms to more challenging angles. The therapeutic goals are to strengthen the upper limb, maintain balance, enhance motor planning, and refine movement timing.

#### Boat Driver

This game prototype relies on the player’s ability to use both hands simultaneously. Using the motion sensing device recognition feature, the avatar (the virtual character portraying the player on the screen) mimics the movements that the player makes. The game starts with the boat in a river, as shown in Figure S14 in Multimedia Appendix 1. The player tries to lead the boat to the goal by moving their hands to steer the boat’s rudder, aiming to reach a specific point within a given time. Visual cues appear on the screen to direct the player’s movement. The game gets more challenging by adding obstacles that the player must avoid before reaching the final destination. The game prototype mechanics focus on improving patients’ speed, endurance, eye-hand coordination, rigidity, ROM, and bilateral movement.

#### Flying for Gold

In this prototype, the player emulates the movement of a flying bird by raising their arms and moving them up and down toward the locations where the golden coins appear. Using the motion sensing device recognition feature, the virtual flying bird on the screen mimics the movements that the player makes. The game starts with the bird appearing on the screen, as shown in Figure S15 in Multimedia Appendix 1, followed by golden coins that appear randomly. The player must collect the coins using the movement mentioned above; the more coins they collect, the higher their score will be. The therapeutic goals are to improve coordination, shoulder movement, and endurance.

#### The Rowing Boat

The prototype is designed to enable the patient to use both arms simultaneously. Using the motion sensing device recognition feature, the avatar (the virtual character portraying the player on the screen) mimics the movements that the player makes. The game starts with the 2-paddled boat in a river (Figure S16 in Multimedia Appendix 1), and the player starts moving his arms (bilateral movement) to reach a specific point at a given time. Visual cues will appear on the screen to guide the player. The faster the player moves the paddles, the quicker they reach the finish line. In the advanced levels, obstacles are introduced, and the player must navigate around them to reach the destination. The prototype mechanics (targeted exercises) focus on improving patients’ speed, ROM, bilateral movement, body awareness, and reducing rigidity.

#### The Steam/Air Pump Game

The game begins with the appearance of a pump and a steering wheel. The objective is for the player to rotate the steering wheel or valve multiple times to release steam, ignite a fire, and cook food within a specified time limit, as illustrated in Figure S17 in Multimedia Appendix 1. Points are awarded when the player turns the steering wheel in the correct direction. Visual cues are displayed on the screen to guide the player’s movements. The game mechanics are designed to enhance movement speed, reduce joint rigidity, improve ROM, and promote bilateral coordination. Figure S18 in Multimedia Appendix 1 shows an alternative scenario within the Air Pump game prototype.

## Results

This section presents the opinions and statements of expert participants regarding the commercial games and game prototypes (mock-ups) that were presented during the focus group. It also includes observations made by the researchers. Therefore, several design implications and recommendations are highlighted.

The participants were excited to test the Kinect-based commercial games, exploring their features and expressing strong enthusiasm for their potential applications. However, the therapist (P4) pointed out that the Kinect Adventures and Motionsports Adrenaline games may not be entirely suitable for patients and would require significant modifications to settings such as speed and difficulty. He added that the extent of a patient’s impairment increases the necessity for customizable difficulty settings. Participants (P20 and P22) who tested the adventure game observed a lack of clear initial in-game guidance, which required the facilitator to provide instructions on how to play. Therapists (P1, P2, P6, P11, and P13) agreed that the mock-up prototypes were effective due to their simplicity and focus on specific movements, whereas the commercial games lacked this focus. It is important to note that these games are typically created for healthy individuals and often provide negative feedback upon losing, which may be unsuitable for patients. However, depending on the patient’s rehabilitation stage and their motor and cognitive abilities, these games can be a fun alternative to conventional rigorous training, particularly for maintaining a training regimen at home after clinical sessions. Participants (P3, P7, and P14) mentioned that in certain cases, the primary goal is to get patients moving and to create a positive experience with motion. Although correct movement is important, it is not always the primary focus. The commercial games have the potential to motivate patients, serving as a foundation for more advanced exercises. However, to be effective, they need adjustable difficulty levels, minimal distracting elements to maintain focus, simplicity, easy navigation, and clear feedback.

Participants (P4, P6, P10, and P12) emphasized that for a game-based rehabilitation system to be effective, therapists’ roles should closely align with their responsibilities in traditional therapy. Once the therapist identifies the therapeutic goals, the game should enable them to assign activities and adjust the exercises to suit the individual impairment characteristics of each patient. In addition, it should enable therapists to track patients’ progress and monitor the quality of their exercise movements.

A participant (P19) highlighted that rehabilitation game systems cannot operate independently (as stand-alone applications). They need to be part of a comprehensive framework that involves patients, therapists, clinicians, hospitals, and other relevant stakeholders, due to the multidisciplinary nature of the rehabilitation process. Participants (P5 and P11) believed that the use of rehabilitation games could help reduce therapists’ workloads by automating exercise movements and providing monitoring and evaluation of patients’ performance. Participants (P17 and P19) suggested expanding the game system to support multiple stations, enabling therapists to work with several patients simultaneously. After each session, patient data would be stored in a database for easy access, review, and performance analysis.

Participants noted that in conventional rehabilitation, therapists used various methods to deliver instructions, including verbal, gestural, and physical approaches. For example, therapists might say, “Move your hand up,” and often combine verbal cues with demonstrations of the movements. When patients had difficulty understanding the instructions through verbal or gestural means, therapists provided physical assistance, guiding the patients through the movements until they understood the instructions. Therefore, the game-based system should use multiple methods to communicate instructions and explain game-related rules to patients, particularly those with cognitive impairments. This includes offering audio and visual instructions through text, images, and animations on the screen. In addition, it is important to provide feedback and multiple visual stimuli during gameplay to help direct and maintain patients’ focus on the required exercises throughout the therapy sessions. In addition, a participant (P17) mentioned that for the game system to be a viable complement to in-hospital rehabilitation, it should provide clear and sufficient instructions for home-based exercises, allowing patients to participate independently. Another participant (19) suggested including video recording functionality to enable therapists to record themselves while playing the game and demonstrate the correct movements for patients to follow at home. Also, participants (P2) proposed that, following a patient’s discharge from the hospital or rehabilitation center, it would be beneficial if the game-based system could record the patient while they practice at home. This would allow therapists to review and evaluate these recordings to monitor the patient’s adherence and ensure the exercises are being performed correctly.

A participant (P15) proposed using an avatar to mirror patients’ movements, as illustrated in the mock-up prototypes, enabling them to observe their actions in real time. To enhance instructional clarity, a second avatar could be included to demonstrate the correct exercise movements.

When asked about the importance of patients seeing themselves and their actions while exercising, as depicted in most of the presented game prototypes, most therapists appreciated the visual representation on the screen. The psychologists (P24 and P25) noted that, for youths and children, using a fun character to demonstrate movements on the screen could be beneficial, as many prefer not to see themselves in real images due to potential negative self-perception. A psychologist (P23) added that role-playing in games is impactful because players develop emotional connections with their avatars. As avatars evolve through different stages, they play a key role in sustaining motivation. Important motivational factors include providing diverse avatar options to boost feelings of autonomy, which in turn fosters positive emotions and connections through the avatar’s development and advancement (P14). In the Reaping the Oranges mock-up game, a participant (P4) recommended updating the avatar to a farmer. This change would let players take on the role of a farmer, harvesting oranges from a tree to fill a basket. He also suggested adding different game scenarios to involve a variety of physical movements. For example, instead of placing the basket beside the player, the avatar could carry a small basket on their head. In this setup, the player would need to reach up to pick oranges from the tree and place them into the basket on their head. A participant (P9) proposed incorporating different game scenarios for subsequent or advanced levels. For example, oranges could appear on a tall tree that the player’s avatar cannot reach by hand. In this case, the avatar could use a small basket, either on its head or held in its hands, to catch the falling oranges before they hit the ground. The avatar would move left or right depending on where the orange will fall, considering the patient’s conditions and limitations (P9). He also suggested that with each new game level, the system could change the shape and size of the tree and add animated objects to the environment to increase visual variety. Participants (P7, P11, and P13) suggested that the game system should allow them to choose from a list of available exercises. This way, each patient can be assigned different exercises based on what the therapist wants to evaluate.

A participant (P23) pointed out that consideration should be taken carefully when choosing game themes, as some themes could trigger negative reactions in patients. For example, a car racing game might be uncomfortable for someone who has been involved in a car accident. To address these concerns, it’s essential to provide a variety of themes to suit different needs and sensitivities. A participant (P15) proposed that farm life could be an excellent theme for game-based physical rehabilitation, and many others (P1, P2, P11, P13, P15, P16, P20, and P22) agreed with this suggestion. This theme could appeal to a broad range of patients, including elderly individuals who may have connections to rural environments from their youth, as well as younger adults and children. The peaceful and relaxing nature of farm life can instill a sense of purpose in patients, potentially improving their commitment to the rehabilitation process. Furthermore, the theme of farm life aligns with the slow pace of seasonal changes and the growth cycles of crops and animals, reflecting the long-term nature of rehabilitation. This theme emphasizes the need for patients to commit to intensive exercise over an extended period, much like the ongoing care required in farming. For example, various farming activities can be adapted into game exercises targeting different movements, such as shearing sheep, herding flocks, milking cows, sowing seeds, planting trees, weeding, fishing, mending fences, cutting grass, picking fruit, harvesting wheat, chopping wood, feeding chickens, selling corn, irrigating, collecting honey, gathering eggs, watering plants, driving a tractor, trimming hedges, spraying pesticides, raking leaves, and scaring birds. A participant (P3) proposed enhancing the game environment by adding a farmer’s house and including more exercises related to activities of daily living (ADLs). Similarly, a participant (P13) emphasized the importance of designing the game system to support the practice of ADL-related movements and tasks. To be effective, game scenarios should replicate real-life activities such as washing dishes, brushing teeth, combing hair, grasping a spoon, holding a cup, shaving, and moving objects. These scenarios should be presented through virtual representations performed by the in-game avatar, incorporating relevant joint movements—such as shoulder abduction and adduction, elbow flexion and extension, and forearm pronation and supination—to interact meaningfully with virtual objects.

A participant (P9) suggested providing a pool of games, each targeting different exercise movements. Another participant (P21) noted that many of the mock-up game prototypes, including The Steam/Air Pump Game, The Rowing Boat, Boat Driver, Colored Boxes, Matching Shapes, Reaping the Oranges, Shuffling Shelves, Organizing Eggs, Rabbit Hunting, and Fallen Apples, could be adapted to fit within a farm life theme under a unified game system. This would provide a variety of game exercises centered around the farm life concept.

Participants (P20 and P23) revealed that the multiplayer aspect of the Reaping the Oranges game, whether through competition, collaboration, or connecting with other patients face-to-face or online, could enhance engagement and enjoyment for many patients. However, it is important to recognize that such interactions may cause stress for some individuals. Therefore, while the game has the potential to enhance the overall experience, it is crucial to consider the diverse preferences and needs of different patients.

A participant (P10) suggested that, given recent technological advancements, it is possible to enable immersive experiences through head-mounted display devices, which are now lightweight and affordable. These devices provide immersive 3D game experiences that can help sustain patient motivation and adherence.

Therapists and other practitioners have been asked about how to transform a physical rehabilitation procedure into a game. It is found that various devices are employed to facilitate therapeutic processes. For instance, during physiotherapy sessions, patients employ various auxiliary devices to facilitate task execution, such as the Shoulder Pulley T Type and the Shoulder Wheel, as depicted in Figure S19 in Multimedia Appendix 1. The shoulder wheel is employed to regain the range of motion in the shoulder joints. Although these devices support the execution of therapeutic movements, they often lack motivational aspects to engage patients. Participants (P3, P10, and P20) noted that integrating gamification with these instrumented devices could fill this gap by turning rehabilitation procedures into interactive and motivating experiences. This approach represents a valuable opportunity for enhancing therapeutic outcomes and deserves further investigation.

Participants (P22, P24, and P14) argued that adapting existing devices used in physiotherapy sessions and transforming them into game controllers could shift patients’ focus from the monotonous repetitions of their exercises to engaging with the game itself. Consequently, actions performed with physical objects in the real world are mapped and reflected in the virtual environment.

The researchers suggested the following game scenarios to demonstrate how these devices can be used: (1) by placing a screen in front of the patients, the Shoulder Wheel can be used to explore a virtual city or world, allowing them to engage with the game and experience tailored stimuli and positive reinforcement. Actions performed by the player using physical objects in the real world are mapped and reflected in the virtual environment. (2) The Shoulder Wheel can also be used for a variety of therapeutic exercises. When operated with both hands, its movements can be integrated into a racing car simulator. Alternatively, when used with one hand, it can be adapted into a firefighter pump game, allowing patients to simulate controlling a water hose to help extinguish a fire. (3) The Shoulder Pulley T Type can be used to simulate the flapping of a bird or the soaring of an aircraft through the skies.

Games based on this setup can be designed to balance patient enjoyment (entertainment value) with an emphasis on the quality of exercise movements (therapeutic value), thereby enhancing both engagement and the effectiveness of the rehabilitation process.

Most participants emphasized that a game-based physical rehabilitation system should offer flexible configuration. It should accommodate diverse cognitive abilities and gameplay preferences by incorporating essential features such as therapy-specific physical movements, adjustable game mechanics, and customizable visual aesthetics.

A participant (P2), who has experience using games in rehabilitation, expressed dissatisfaction with the preprogrammed difficulty levels in games such as adventure games. The participant emphasized the need for more flexible control over game parameters, allowing therapists to adjust the difficulty to suit the individual needs and progress of each patient.

A participant (P16) suggested that developers could take advantage of existing commercial games to create diverse game scenarios. He added that a game like Kinect Adventures: 20,000 Leaks could be modified by simplifying its design, enabling therapists to control aspects such as difficulty level, speed, and range of motion, and to tailor patients’ interactions with virtual game objects.

A participant (P14) argued that the game prototypes presented in the Intervention Using Low-Fidelity Paper Mock-Ups section are designed to permit only predefined exercise movements to interact with or play a specific game. For example, in the Blowing the Bubbles game, patients use reaching movements to blow the bubbles. In the Boat Driver game, patients perform circular movements to control the boat. A game system that enables therapists to customize and align different physical movements with game actions (such as using circular motions to maneuver a jet fighter) can enhance the gaming experience for patients and support their rehabilitation goals more effectively.

Most participants believed that a game system with reconfigurable visual elements, tailored to patients’ preferences and needs, can enhance their engagement and participation in game-based therapy.

Participants (P4, P7, P22, and P24) noted that patients may react differently to the visual design of rehabilitation games. Individuals with cognitive impairments often struggle to process highly realistic graphics, which can lead to disengagement. In such cases, simplified 2D or cartoon-like visuals may improve comprehension and promote participation in therapy. In addition, older patients or those with negative attitudes toward gaming may view these games as childish and lacking therapeutic value. To address this concern, a participant (P15) suggested that game designs could be adjusted to reflect real-world therapeutic activities, thereby reinforcing their role as serious tools for rehabilitation. Therefore, a game-based system should be able to adjust its visual settings and reconfigure its appearance to match a patient’s preferences and needs, as this is crucial for enhancing engagement and participation in game-based therapy. This highlights the need for future research to develop tools that enable therapists to adjust and tailor games to individual patients’ preferences and needs.

On the other hand, the controllers of the rehabilitation game system should be designed to enable patients to practice various exercise movements while intentionally suppressing their compensatory behaviors. A participant (P11) suggested that the controller could be configured by the therapist to recognize only the horizontal movements of the patient’s hand, or to consider trunk movements as input, thereby reducing trunk-related compensatory actions. Likewise, for stroke patients who unintentionally involve their shoulder or elbow, the game system can allow the therapist to address this by using the compensating joint as an input. Furthermore, the system should be adjustable and flexible, allowing the therapist to choose which hand or side of the patient (left or right) will be used for game interaction.

A participant (P5) suggested implementing a dedicated user interface for therapists to enable efficient control of the game system. This interface would allow therapists to configure game settings. For instance, if a patient begins to show compensatory behaviors during gameplay, the therapist can adjust the game settings to reduce the range of motion or slow down the game pace. This allows the patient to perform movements without severe compensatory behaviors. When patients experience fatigue or pain, therapists can make the games less challenging or pause them. In addition, therapists can have full control over the games to maintain a balance between therapeutic and engagement values.

Another participant (P6) argued that in conventional rehabilitation sessions, therapists must physically interact with patients to correct inappropriate movements. This often requires therapists to stand or sit next to or behind their patients, meaning they need to continuously reposition themselves throughout the therapy sessions. Thus, a dedicated and portable user interface is essential, allowing therapists to control game-based therapy more efficiently using handheld devices like tablets or mobile phones. This interface would facilitate better interaction with the games and improve the moderation of therapy sessions.

Consequently, based on the earlier discussion, it is evident that there is a need for game systems with high configurability. Such systems would allow therapists to customize therapy programs to better meet individual patient needs. These systems should offer the flexibility required for therapists to tailor therapy programs to each patient’s specific needs. However, the increased complexity of game system operations places additional demands on therapists and requires comprehensive training to effectively manage and moderate game-assisted therapy sessions.

Therapists and other practitioners emphasized the importance of proper training to effectively utilize rehabilitation games as a therapeutic tool. For instance, a participant (P1) remarked, “I am motivated to use games with my patients, but I don’t know what to do.” Therapists explained that improper use of rehabilitation games can result in patient pain or injury, making it challenging for therapists who are new to or lack experience with game-based therapy. Participants (P22 and P25) noted that using rehabilitation games without previous training can make it difficult to determine how to tailor the therapy to meet patients’ needs effectively. With proper training, therapists would gain a clearer understanding of their roles, leading to more efficient use of the games and an improvement in the quality of game-based therapy sessions. Therefore, thorough training on the game system is crucial for therapists to let them grasp the system’s details and learn the most effective ways to use it. Consequently, it is essential to thoroughly assess and prioritize the usability of the game system for therapists to ensure it is both effective and user-friendly. This underscores the necessity for future research to examine the usability and effectiveness of interactive mechanisms that allow therapists to configure and customize game-based interventions.

On the other hand, therapists revealed that the data collected from the game-based intervention can provide various insights. While the scores at the end of a game session are commonly used to measure performance, additional metrics can be gathered to offer insights into patients’ motor and cognitive abilities. These include the following: (1) score: higher scores may indicate better performance in response to challenging tasks; (2) time: longer session durations suggest a greater number of repetitions of the prescribed exercises; (3) level: advancing to higher game levels is associated with increased challenges; (4) distance: Assessing the distance covered by the affected limbs helps determine the level of muscle engagement and effort exerted during the exercise; (5) changes in motion direction: a higher frequency of changes in movement direction might indicate inaccuracies in execution or that the movements were performed without a clear purpose; (6) initial movement range/amplitude: this metric is crucial for evaluating patients’ progress and understanding their development over time; and (7) number of sessions: tracking the number of sessions played and any interruptions can help therapists determine whether patients felt uncomfortable with specific games or if they were disengaged and did not complete the prescribed exercises. In addition, one therapist mentioned that electromyography (EMG) sensors could be used to monitor muscle activity during exercises. By recording EMG readings, therapists can assess movement patterns, measure muscle fatigue, identify disorders, and evaluate the effectiveness of exercise interventions.

Movement correction measure: A key factor in the effectiveness of game-based rehabilitation systems is the accurate assessment of patient movements during gameplay sessions. However, according to a participant (P13), the measures taken to prevent incorrect execution of exercises in motion-sensing technology–based games are limited, as patients at risk of injury are typically not permitted to use motion-tracking systems. It remains the therapist’s responsibility to evaluate and manage these situations. Another participant (P5) highlighted that some of the major benefits of using this technology include engaging patients, making exercise enjoyable, providing opportunities for social interaction, and offering feedback on exercises. The experts were less worried about the games enforcing or ensuring correct movement and addressing compensatory actions, as they would personally guide and decide when and how patients should use motion games.

According to the discussed metrics, these metrics are expected to provide valuable insights for therapists to evaluate a patient’s recovery progress. For instance, low scores and extended playtimes may indicate that patients are struggling to complete the required tasks, which can result in frustration and a negative perception of the game. In addition, covering longer distances with the affected limb and making fewer changes in motion direction suggests that patients are performing movements with greater amplitude, indicating they are in a more advanced stage of recovery. Conversely, if patients cover short distances with frequent changes in motion direction, it indicates that they either struggle to perform wide gestures or exhibit inaccuracies in their movements. Thus, while the patient enjoys the game, these parameters can be collected and stored for later analysis. This allows therapists to monitor and assess the patient’s progress and development over time, providing insights into the quality and effectiveness of the game exercises performed. Another therapist suggested developing an online game-based system accessible to both patients and therapists. Therapists would log in to the system to view their patients, and once a specific patient is selected, the therapist could access their records, review collected data, and customize each game’s settings as needed to better suit the patient’s needs.

## Discussion

### Principal Findings

The observations, feedback, and suggestions from the focus group were analyzed, leading to the development of several guidelines to inform the design and development of an effective game-based physical rehabilitation system. To increase compliance and ensure patients perform their exercises correctly, the game must maintain patient engagement and provide support during exercises. This can be achieved through appropriate feedback. Instant and clear feedback is crucial for providing a meaningful gameplay experience. It helps patients understand the outcomes of their actions, reflects their performance in exercises, and offers various in-game achievements. Feedback should include visual, audible, and haptic elements to enhance the user’s interpretation of the gameplay and maintain their engagement. In addition, a game-based system needs to facilitate the practice of movements and tasks related to ADLs.

The social aspect of the game system is crucial, enabling patients to play with family members, friends, or other patients. Highlighting this feature enhances the overall experience. In addition, incorporating a tracking mechanism for the quality of the patient’s movements in the game system is advantageous. This will effectively support and facilitate the rehabilitation process, encouraging patients to perform the exercises accurately. In addition, a potential game-based system should be capable of tracking patients’ progress. This feature could benefit patients by providing positive reinforcement and encouraging compliance and also assist physiotherapists by enabling them to monitor and assess patient development. In addition, simple art styles and graphics, as seen in the mock-up prototypes, can motivate players and make the games more approachable, a feature that participants seemed to appreciate. This cartoon-like, simplistic art style has benefits such as lower processing power requirements and encouraging a more playful state of mind compared to realistic graphics. However, different demographics might respond differently to this style, as many people today are accustomed to games with nearly photo-realistic graphics, such as Call of Duty. People generally tend to prefer familiar styles. On the other hand, since rehabilitation programs typically involve multiple exercises, the game system should be designed to incorporate them. Therefore, the game system should be expandable to include a variety of exercises, supporting the entire rehabilitation process.

Furthermore, the game system should offer significant flexibility and adaptability to suit each patient’s specific degree of injury. It is essential for a potential game-based rehabilitation system to allow therapists to customize the game exercises. Since individuals have different needs based on their injury and stage of rehabilitation, customization ensures that the game can better accommodate these unique patient requirements and preferences. Given that the rehabilitation process often extends over a long period, the focus of the training changes throughout this time. Therefore, the game system should include mechanisms for adapting game interventions to accommodate the different stages and focuses of rehabilitation. On the other hand, ensure that patients can concentrate on their exercises rather than dealing with technical details. The findings highlight the need for a rehabilitation game to be simple to install and start. It should be easy to begin without lengthy or complex setup processes. For home use, the game system should prioritize easy installation, fast start-up, and intuitive usability. Table 2 presents a summary and categorization of the design guidelines for game-based physical rehabilitation systems.

**Table 2.** Design guidelines for the game-based physical rehabilitation system.

Category	Guidelines
Gameplay design and usability	Support simplified gameplay mechanics to enhance usability and accommodate patients with varying cognitive and physical abilities. Integrate multimodal instructional methods, including audio, visual, text, and animation, to support understanding of game mechanics and therapeutic tasks. Include accessibility features such as simplified controls, multiple visual modes, easy-to-navigate menus, and customizable interface styles to support diverse user needs, allowing the system to cater to different user preferences, cognitive loads, and therapeutic requirements. Support game-based exercises tailored to the cognitive and physical capacities of patients. Provide instant feedback and multiple visual stimuli during gameplay to guide and sustain patients’ focus on the required exercises. Use positive reinforcement and reward mechanisms to motivate patients and enhance engagement during therapy. Support home-based exercise modules that are self-explanatory and include clear, structured instructions to enable independent participation. Embed clear and intuitive tutorials, supported by visual and auditory cues, as well as explicit in-game guidance, to encourage independent use and reduce the need for external support. Provide optional multiplayer modes to promote social interaction and engagement when appropriate for the therapeutic context.
Game themes and scenarios	Provide a variety of themes and scenarios to suit different needs and sensitivities. Avoid game themes that may trigger distress. Themes should be carefully selected to prevent negative reactions (eg, racing games for car accident survivors) and instead incorporate neutral, calming settings (eg, farm life with seasonal progression) that align with rehabilitation goals. Incorporate activities of daily living (ADLs), such as fruit picking, brushing teeth, and combing hair, to enhance task relevance and promote functional recovery. Use stylized, age-appropriate avatars rather than live camera images of patients to enhance comfort and engagement during gameplay. Offer diverse avatar choices and thematic avatars matching game contexts. Incorporate avatar customization and progression systems and offer upgrade options to enhance autonomy and sustain patient motivation throughout therapy. Implement a dual-avatar system, where one avatar mirrors the patient’s movements and another, representing the instructor or therapist, demonstrates the correct form to facilitate self-correction.
Therapeutic focus and therapist control	Implement therapist interfaces that allow dynamic adjustment of exercise parameters such as speed, difficulty, and range of motion during rehabilitation sessions. Enable therapists to adjust difficulty, customize visuals, and adapt game mechanics to accommodate patient limitations and cognitive impairments. Allow therapists to target specific motor functions by selecting exercises from a game-based exercise list and assigning them based on individual patient impairments and needs. Provide a portable, handheld (tablet or phone) interface for therapists to seamlessly control sessions, including real-time form alerts, emergency stop, pause, and override functions to ensure patient safety, correct movement, and efficient session management. Include tools that allow therapists to translate and map physical rehabilitation movements into game mechanics, ensuring clinical relevance and effectiveness. Enable the therapist to define movement constraints: the system should be adjustable and flexible, allowing the therapist to choose which hand or side of the patient (left or right) will be used for game interaction. Provide video recording functionality for therapists to demonstrate exercises remotely. Enable multiuser support, centralized patient databases, and access for multiple stakeholders or practitioners.
Movement quality, monitoring, and data management	Incorporate features to track performance metrics, including scores, time, directional changes, movement precision, range of motion, limb engagement, muscle activation, and compensatory movements. Implement AI-assisted compensatory movement detection with real-time alerts to prevent patients from persisting through pain or incorrect form. Store data for longitudinal analysis to inform therapy adjustments. Integrate motion tracking with electromyography (EMG) data to enable comprehensive monitoring of muscle activity during exercises. Include postsession analytics and progress monitoring dashboards. Develop comprehensive dashboards for therapists that display and visualize patient progress, compensation behaviors, adherence levels, and movement quality trends over time.
Technology and device integration	Support the integration of virtual reality or augmented reality and diverse interaction technologies to enhance the effectiveness and engagement of rehabilitation experiences. Support the integration of EMG biofeedback to track muscle activation, fatigue thresholds, intervention efficacy, and exercise effectiveness. Gamify existing rehabilitation devices, such as shoulder wheels and pulleys, by transforming them into game controllers that map real-world movements to virtual environments. This allows patients to navigate and interact with the game, thereby enhancing the effectiveness of rehabilitation. Implement an online platform that supports secure data access and remote configuration.

### Limitations

Therapists and health care professionals were actively involved in this phase of the study due to their critical role in implementing effective game-based rehabilitation. Although the inclusion of brain injury survivors (including those with traumatic injuries and stroke) is equally significant, it was beyond the scope of this paper. Their perspectives and participation will be incorporated into future phases of the research and addressed in subsequent publications.

### Conclusions

Incorporating serious games and advances in information and communication technologies into physical rehabilitation can enhance the rehabilitation process, offer benefits beyond those of conventional methods, and improve both the effectiveness and efficiency of rehabilitation. The aim is to (1) enhance the effectiveness of game-based therapy for patients who struggle to engage with or benefit from it, and (2) assist therapists by offering a game-based rehabilitation system tailored to their needs. To investigate the factors, determinants, requirements, and actual needs for potential game-based rehabilitation, a focus group with therapists and other relevant health care professionals is organized. This decision aims to evaluate the ideas with real rehabilitation therapists and practitioners. To effectively discuss the ideas with therapists and other related professionals before starting development, available commercial games are tested, and various paper game mock-ups are evaluated. This approach facilitated productive discussions, validated the direction, and is expected to optimize our development timeline. The focus group discussions yielded several guidelines that will serve as the foundation for the next phase of our research. This phase will involve the design, development, and implementation of a game-based physical rehabilitation system aligned with these guidelines.

## Supplementary material

10.2196/67336 Multimedia Appendix 1 Illustration of gameplay.

## Abbreviations

ADL: activity of daily living
EMG: electromyography
ROM: range of motion
