# Supplementary material for: Design Guidelines for a Game-Based Physical Rehabilitation System: Focus Group Study
Source: JMIR Hum Factors. 2025 Sep 26;12:e67336. doi: 10.2196/67336 (PMC12468163; doi:10.2196/67336)
Supplement: Multimedia Appendix 1 [file humanfactors-v12-e67336-s001.pdf]

Multimedia Appendix

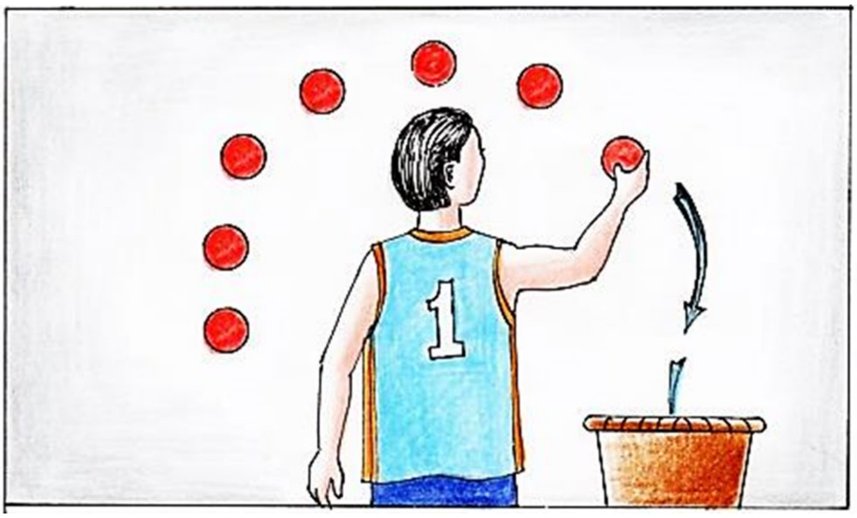

Figure S1. Rabbit Hunting game prototype.

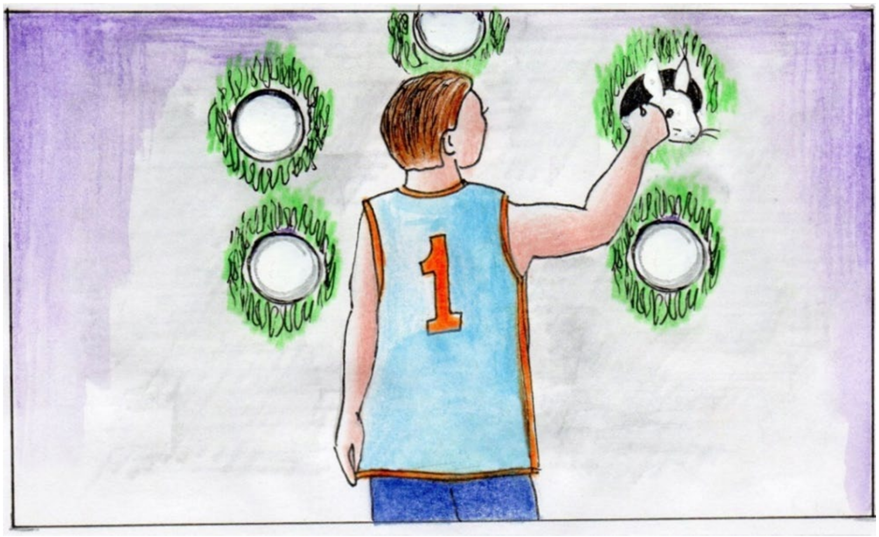

Figure S2. First level of the Picking the Balls game prototype.

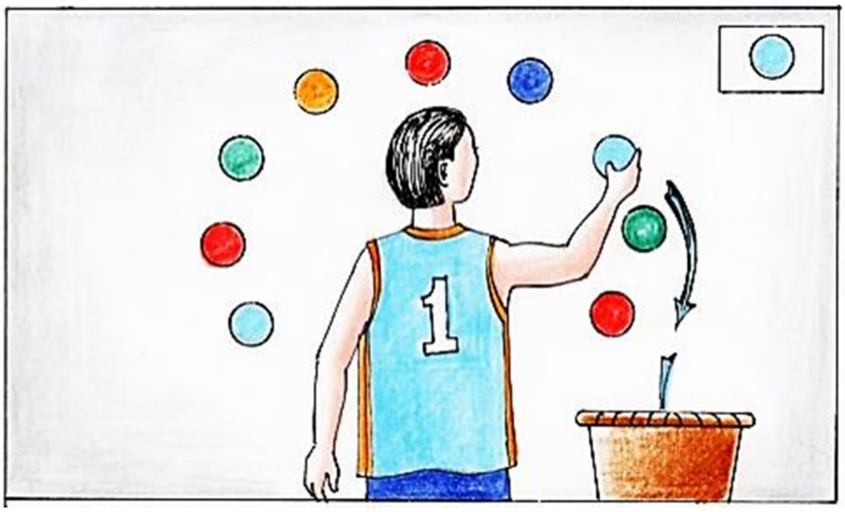

Figure S3. Second level of the Picking the Balls game prototype.

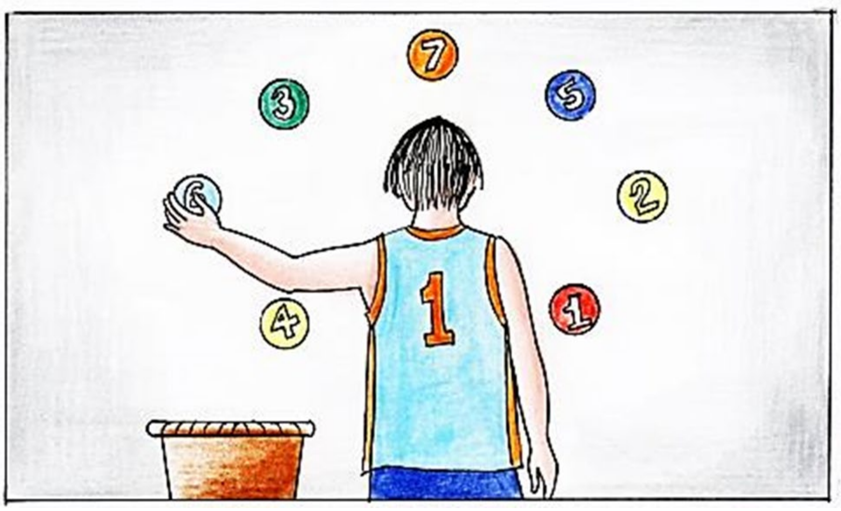

Figure S4. Final level of the Picking the Balls game prototype.

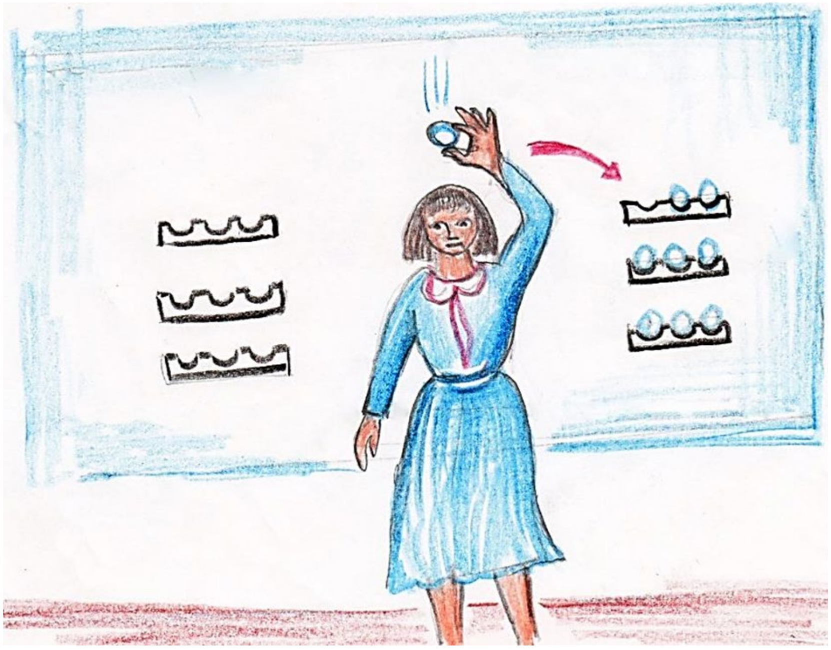

Figure S5. Organizing Eggs game prototype.

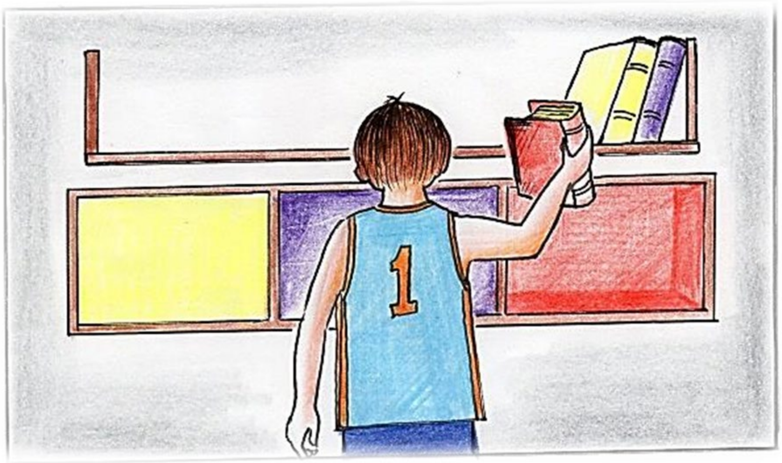

Figure S6. Shuffling shelves game prototype.

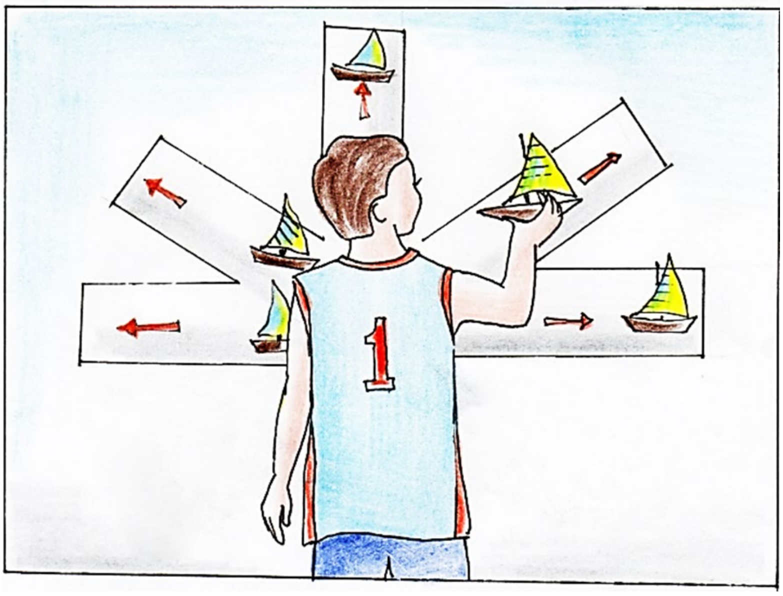

Figure S7. Hold the Boat game prototype.

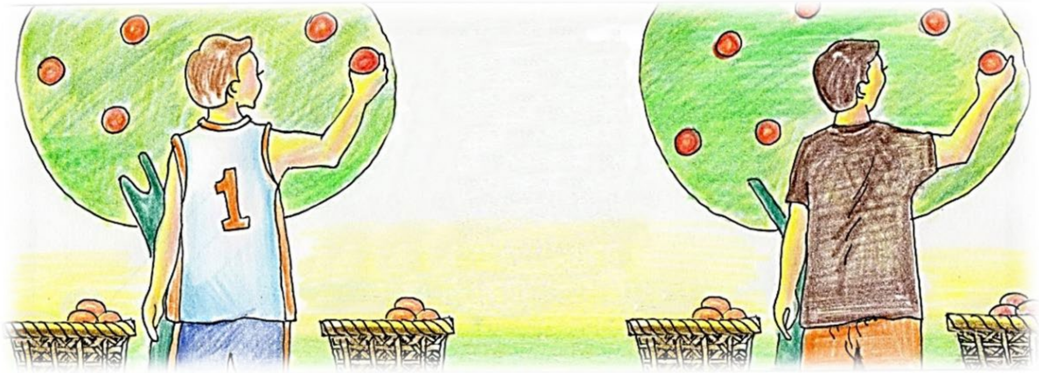

Figure S8. Grabbing the Oranges game prototype.

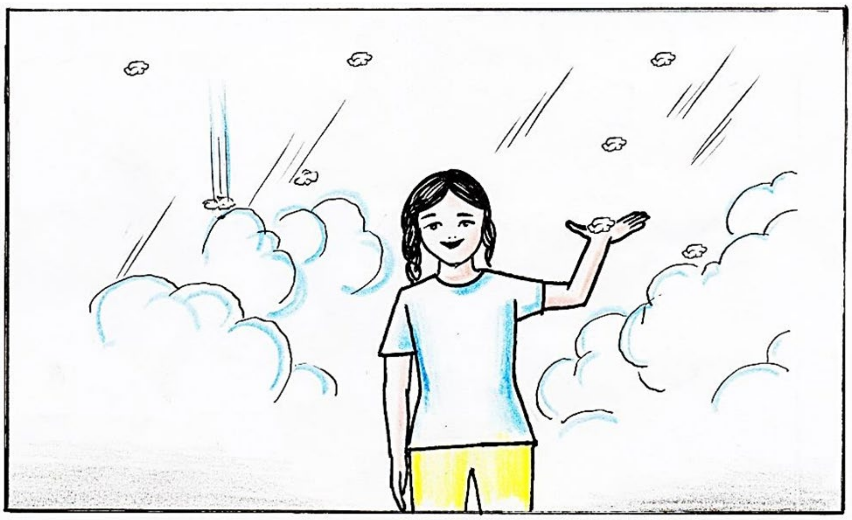

Figure S9. Ice Ball game prototype.

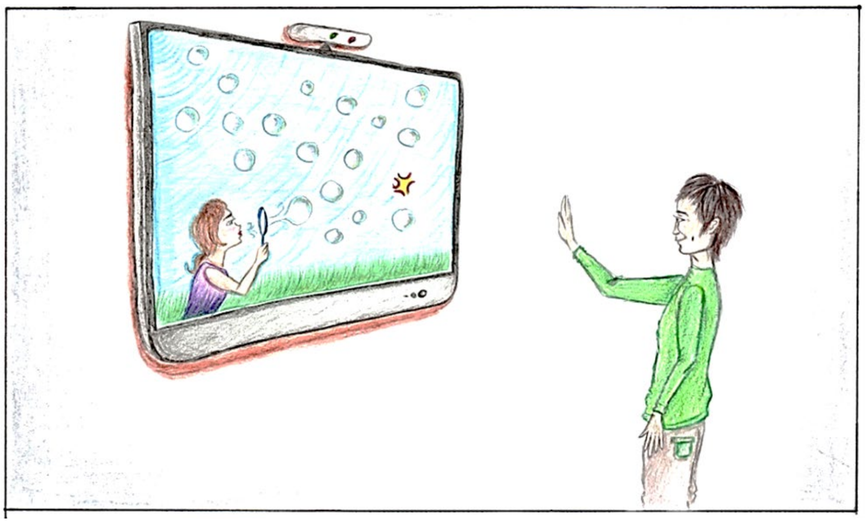

Figure S10. Blowing the Bubbles game prototype.

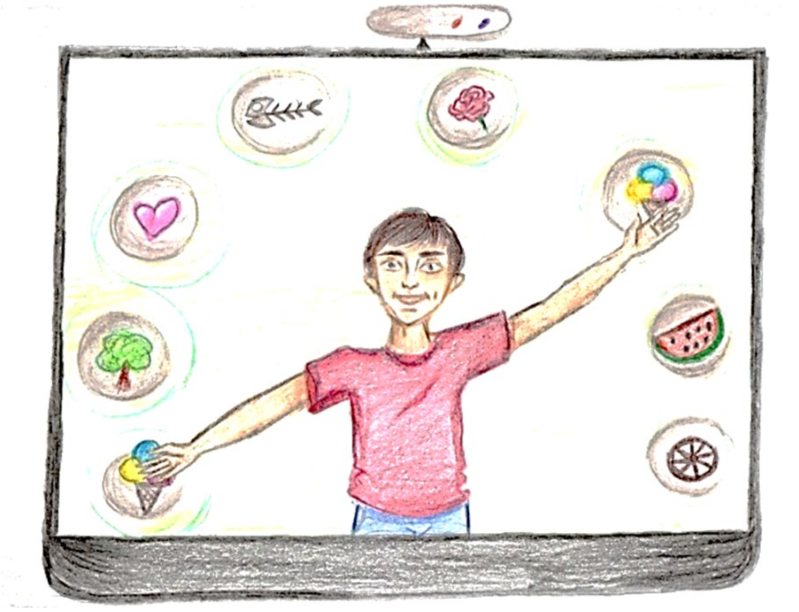

Figure S11. Matching shapes game prototype.

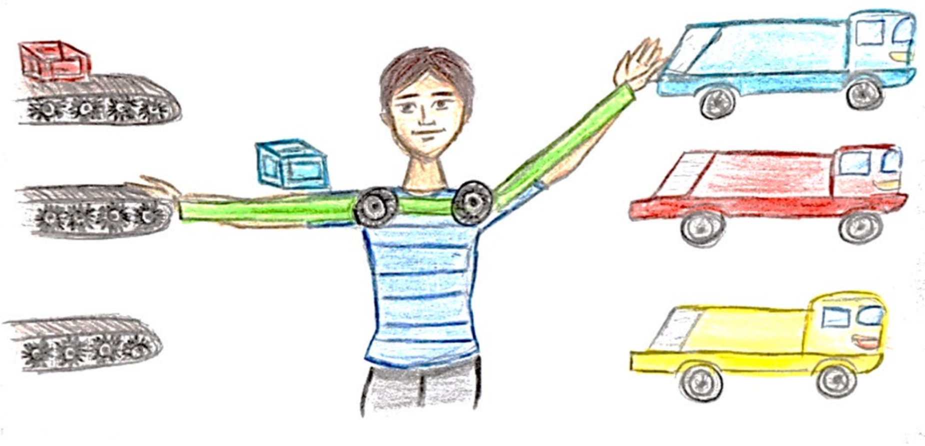

Figure S12. Colored boxes game prototype.

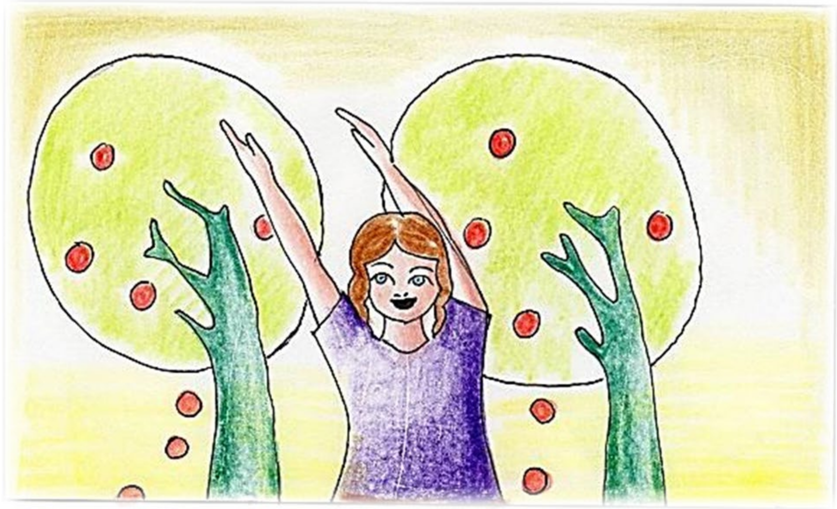

Figure S13. Fallen Apples game prototype.

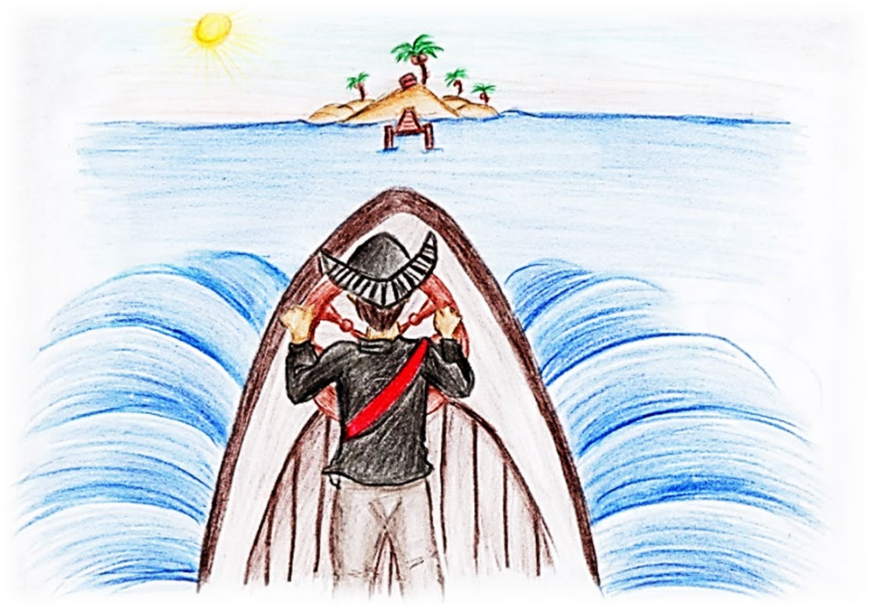

Figure S14. Boat Driver game prototype.

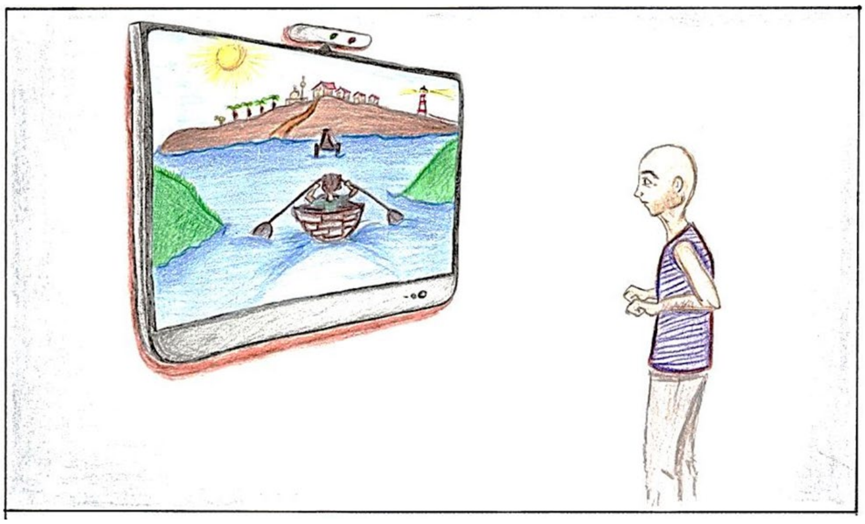

Figure S15. The Rowing Boat game prototype.

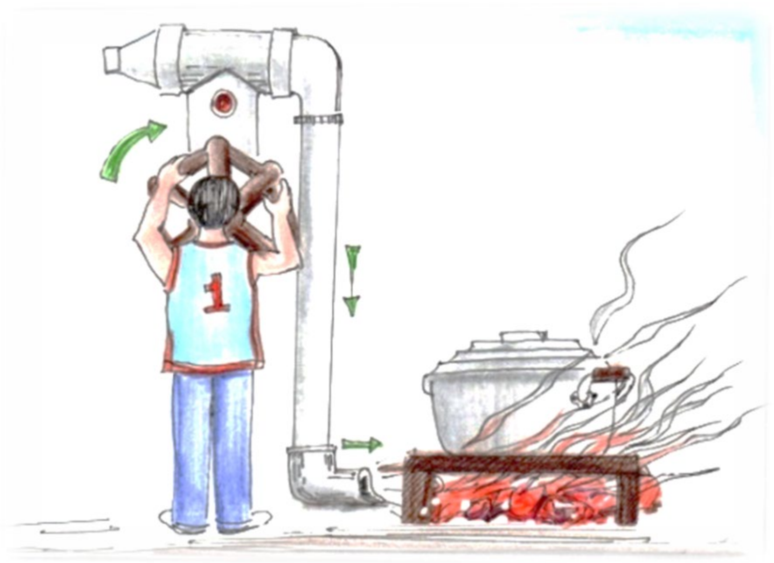

Figure S16. Air Pump game prototype.

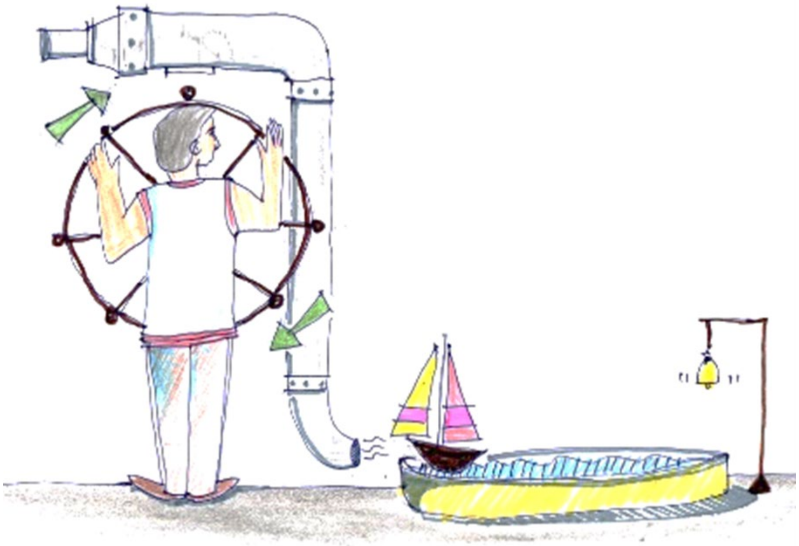

Figure S17. A different game scenario in the Air Pump game prototype.

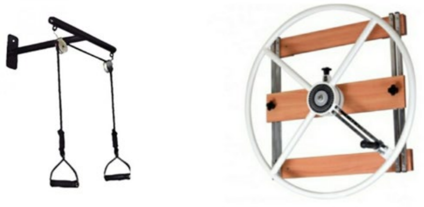

Figure S18. Shoulder Pulley T-Type and Shoulder Wheel, displayed from left to right as examples of physiotherapy equipment.
